# Profiling of Bioactive Metabolites and In Vitro Multifunctional Properties of Postbiotic Cell-Free Supernatant Derived from Probiotic Lactic Acid Bacteria Isolated from Fermented Olives

**DOI:** 10.3390/ijms27209138

**Published:** 2026-10-15

**Authors:** Houssam Abouloifa, Imane Maiouet, Asma Bouqafa, Nada Mouhtadi, Moussa Bouzzit, Nour Eddine Bentouhami, Nabil Ghabbour, Fouad Mellouki, Salwa Karboune

**Affiliations:** 1 Research Unit of Microbiology, Biomolecules, and Biotechnology, Laboratory of Chemistry-Physics and Biotechnology of Molecules and Materials, Faculty of Sciences and Techniques—Mohammedia, Hassan II University of Casablanca, Casablanca 28806, Morocco; imanemaiouet@gmail.com (I.M.); bou18098@gmail.com (A.B.); nada.mouhtadi@hotmail.com (N.M.); moussabouzzit4@gmail.com (M.B.); fouad.mellouki@fstm.ac.ma (F.M.); 2 Laboratory of Bioresources, Biotechnology, and Sustainable Development (L2BD), Faculty of Sciences, Mohammed Premier University, Oujda 60000, Morocco; noureddine.bentouhami@ump.ac.ma; 3 Laboratory of Natural Resources and Environment, Faculty of Applied Sciences of Taza, Sidi Mohamed Ben Abdellah University, B. P. 1223, Taza 35000, Morocco; nabil.ghabbour@usmba.ac.ma; 4 Department of Food Science and Agricultural Chemistry, Macdonald Campus, McGill University, 21111 Lakeshore, Sainte-Anne-de-Bellevue, QC H9X 3V9, Canada; salwa.karboune@mcgill.ca

**Keywords:** lactic acid bacteria, postbiotic-CFS, bioactive metabolites, multifunctional, health

## Abstract

This work aims to characterize the bioactive metabolite profile and in vitro multifunctional properties of postbiotic cell-free supernatants (Postbiotic-CFSs) derived from three lactic acid bacteria (*Levilactobacillus* brevis S27, *Lactiplantibacillus pentosus* S42, and *Lactiplantibacillus plantarum* S62) isolated from fermented green olives. The postbiotic-CFSs were analyzed for their bioactive metabolites, emulsification index E24%, and antimicrobial, antioxidant, anti-inflammatory, and antidiabetic activities. The bioactive metabolites identified in all postbiotics included organic acids (lactate, citrate, and oxalate), SCFAs (acetate), hydrogen peroxide, diacetyl, exopolysaccharides (EPSs), proteins, biosurfactants, polyphenols, and flavonoids. The postbiotic-CFSs exhibited emulsification index (E24%) values ranging from 21.5% to 38.2%. Furthermore, they demonstrated antimicrobial properties with inhibition diameters ranging from 14.10 to 16.9 mm against pathogenic bacteria and from 15.8 to 23.50 mm against yeast, while fungal mycelial growth was inhibited by 35.77 to 45.09%. All postbiotics demonstrated antioxidant activities of DPPH from 65.91 to 68.55% and FRAP values from 1707.31 to 1765 µg AAE/mL, as well as anti-inflammatory activity (11.83–12.88%) and antidiabetic activity (34.38–93.75%). These findings highlight the significant potential of postbiotic-CFSs as natural bio-preservative agents in food products and as a source of bioactive metabolites with multifunctional health benefits for consumers.

## 1. Introduction

Lactic acid bacteria (LAB) are a group of Gram-positive cocci or rods, a type of non-spore-forming, non-respiring bacteria responsible for producing lactic acid through the fermentation of carbohydrates [1], and they demonstrate diverse roles and applications in food, gut health, biomedicine, agriculture, and the environment [1,2]. Thus, LAB are generally recognized as safe (GRAS) and show beneficial health properties, leading them to be considered probiotics [3]. The FAO and WHO define probiotics as “live microorganisms which, when administered in adequate amounts, confer a health benefit on the host”. According to the current definition, probiotics are required to be living microorganisms, excluding non-viable bacterial cells or their components. Nevertheless, research has shown that inactivated probiotic bacteria can still exert biological effects on the host and are considered postbiotics.

Postbiotics are inanimate microbial cells, cell fragments, or metabolites produced during fermentation [4]. They are also known as metabolites, cell-free supernatants (CFSs), or bioactive compounds generated by live probiotics or liberated following bacterial lysis [5]. Moreover, a CFS is an inanimate liquid preparation derived from bacterial fermentation that contains active metabolites, provides beneficial effects, and is considered a postbiotic [6]. The precise definition of postbiotics, according to the International Scientific Association for Probiotics and Prebiotics (ISAPP), is defined as a “preparation of inanimate microorganisms and/or their components that confers a health benefit on the host” [7], and they are a promising, stable, and effective alternative to traditional probiotics and prebiotics [4]. Furthermore, several recent studies classify sterilized CFSs containing microbial metabolites and cellular components as postbiotics when evaluating functional properties [8,9]. In this study, the postbiotic preparation comprises a bioactive cell-free supernatant (CFS) containing secreted cellular components and metabolites produced during fermentation.

Postbiotics exhibit various functional properties and innovative applications across multiple sectors, including human nutrition, animal feed, and the food, pharmaceutical, and cosmetic industries [10,11,12]. Moreover, postbiotics present a collection of compounds including probiotics, such as polymeric macromolecules (exopolysaccharides (EPSs) and peptides), low-molecular-weight metabolites (short-chain fatty acids (SCFAs), organic acids, and volatile compounds), cell-free supernatants (CFSs) (peptides, reuterin, hydrogen peroxide, phenolic compounds, cyclic dipeptides, biosurfactants, and flavonoids), and structural cell-wall components (peptidoglycan, teichoic acids, and lipoteichoic acids) [13]. Furthermore, postbiotics derived from bacteria demonstrate various therapeutic properties, including antibacterial, antioxidant, anti-inflammatory, antidiabetic, anticancer, and immunomodulatory activities [14,15,16]. They also provide natural antimicrobial compounds against pathogenic fungi [17,18], applied in the development of food antimicrobial packaging to extend food shelf life [19,20], and are used as functional ingredients in food products [12].

In recent years, research on postbiotics has increased and garnered significant attention due to their many advantages. In this context, our previous study focused exclusively on isolating antifungal LAB and characterizing the probiotic properties (e.g., acid/bile tolerance, hydrophobicity, auto-aggregation, and direct antifungal activity) of the viable LAB strains from fermented green olives. In this study, we aimed to demonstrate various bioactive compounds in postbiotic cell-free supernatants derived from lactic acid bacteria strains and to investigate in vitro multifunctional properties (antimicrobial, antioxidant, anti-inflammatory, antidiabetic, and emulsifying activities) of these novel postbiotic cell-free supernatants.

## 2. Results

### 2.1. Profiling of Bioactive Metabolites from Postbiotic-CFS

**Figure 1:**
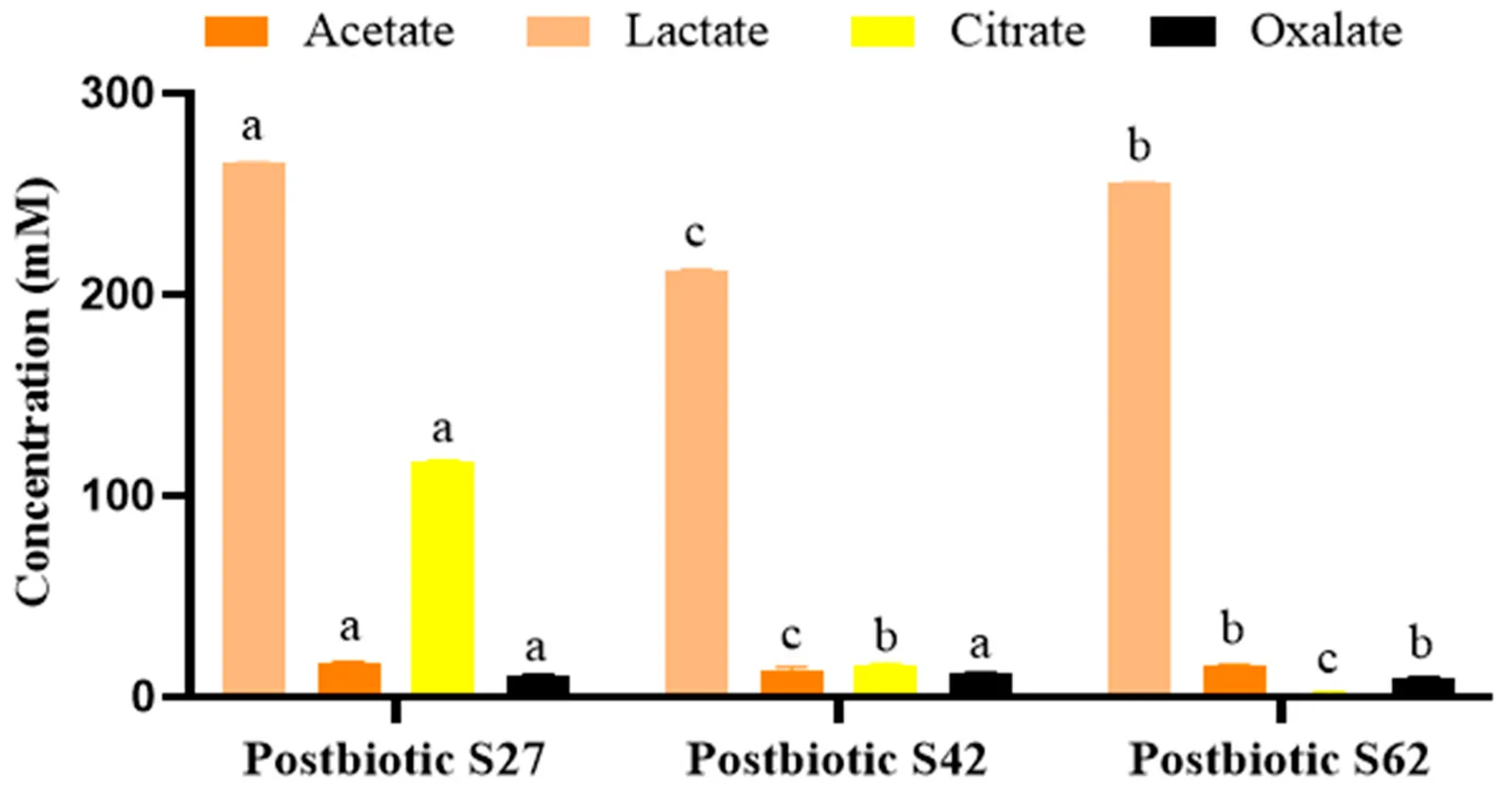
Organic acids and short-chain fatty acid production in postbiotic-CFSs derived from *Levilactobacillus* and *Lactiplantibacillus* strains. ^a,b,c^ Means in the same postbiotic-CFS at each parameter with different lowercase letters differed significantly (*p* < 0.05).

**Figure 2:**
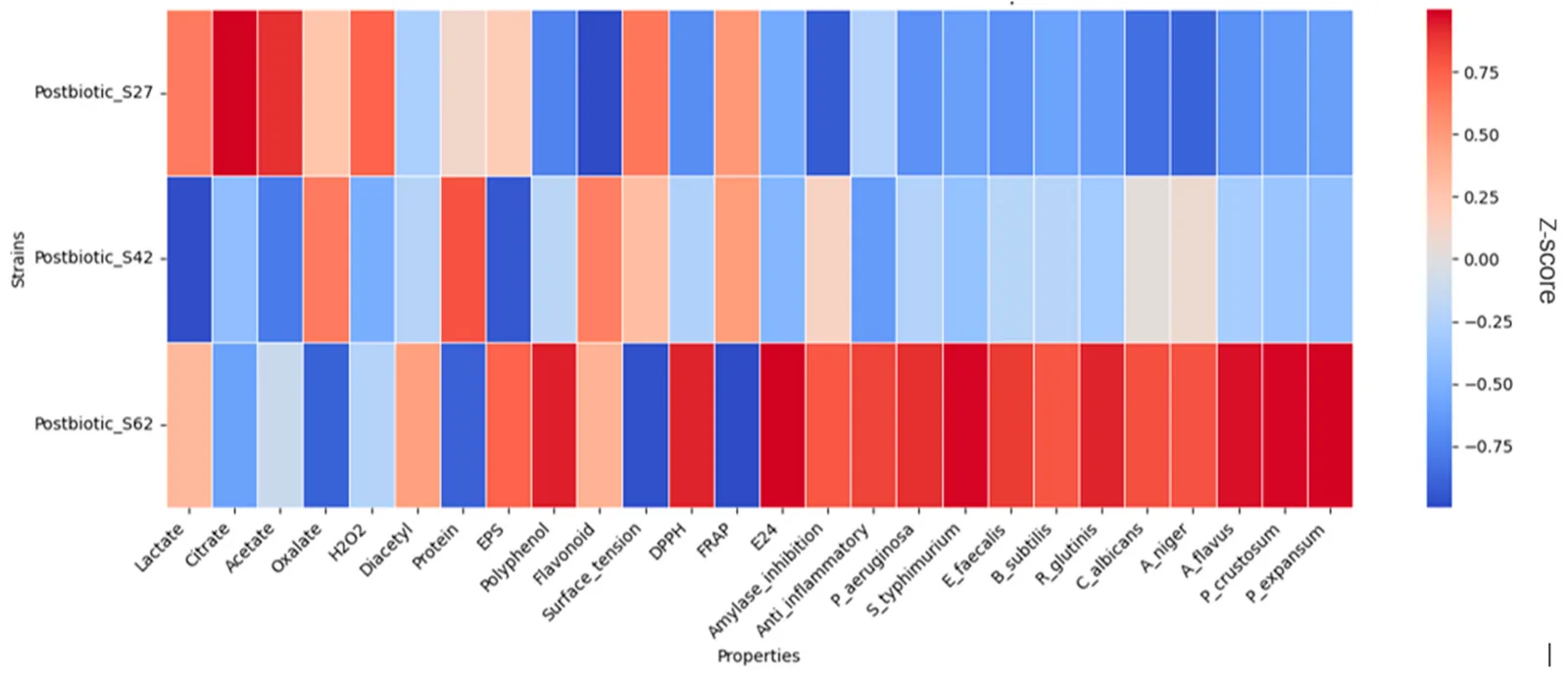
Heatmap of the association between postbiotic-CFSs derived from *Levilactobacillus* and *Lactiplantibacillus* strains and their bioactive profiling and functional properties. Color intensity reflects the normalized feature value (z-score) for each parameter across strains.

The characterization of the metabolite profile of postbiotics was performed using modern instruments and colorimetric methods. The results of the organic acid and short-chain fatty acid (SCFA) quantification by HPLC in the postbiotics from *Levilactobacillus* and *Lactiplantibacillus* strains are indicated in Figure 1. Lactate, citrate, and oxalate are organic acids, while acetate is a short-chain fatty acid (SCFA). Organic acids and short-chain fatty acid (SCFA) profiles across all postbiotics were significantly (*p* < 0.05) dominated by lactate, accompanied by lower concentrations of citrate, acetate, and oxalate. Lactate was the predominant organic acid produced in postbiotic-CFS S27, postbiotic-CFS S42, and postbiotic-CFS S62, with concentrations of 265.46 mM, 211.82 mM, and 255.28 mM, respectively. Citrate was produced at a high concentration (116.92 mM) in postbiotic S27. Moreover, low concentrations of acetate and oxalate were produced in all postbiotics, ranging between 13.73–17.49 mM and 9.30–11.53 mM, respectively. Acetate concentrations detected in postbiotic-CFSs were lower than the initial concentration in uninoculated MRS broth, indicating metabolic consumption of acetate by *L. brevis* S27, *L. pentosus* S42, and *L. plantarum* S62 during fermentation. Conversely, postbiotic-CFS S27 exhibited a high citrate concentration, which may be due to citrate synthesis and accumulation or co-elution of polar compounds. The results of other metabolites produced are presented in Table 1. The protein contents of postbiotic-CFSs S27, S42, and S62 were 177.16 µg/mL, 192.42 µg/mL, and 154.65 µg/mL, respectively. Moreover, the exopolysaccharide (EPS) concentrations were 535.27 mg/L, 244 mg/L, and 600.62 mg/L in postbiotic-CFSs S27, S42, and S62, respectively. The hydrogen peroxide and diacetyl concentrations were detected in low concentrations, 0.0070–0.0074 µg/mL and 0.43–0.59 µg/mL, respectively (Table 1), and no significant difference (*p* > 0.05) was observed among all postbiotic-CFSs. On the other hand, Polyphenol and flavonoid concentrations were 386.00 µg GAE/mL, 407.60 µg GAE/mL, and 451.93 µg GAE/mL; and 83.66 µg QuE/mL, 114.51 µg QuE/mL, and 109.73 µg QuE/mL in postbiotic-CFSs S27, S42, and S62, respectively (Table 1). Furthermore, an investigation into biosurfactant production was conducted using several methods, and the results are summarized in Table 2. The results showed the ability of all strains to produce biosurfactants in all postbiotics, based on an oil displacement test with diameters between 7.1 mm and 10.3 mm, a Parafilm M Test, a drop collapse test (microliter plates), and a glass slide test (Table 2). In addition, the surface tensions of postbiotic-CFS S27, S42, S62, and uninoculated MRS are 38.3 mN/m, 37.45 mN/m, 34.35 mN/m, and 40.65 mN/m, respectively, indicating a higher biosurfactant concentration in postbiotic S62 compared to postbiotic-CFSs S27 and S42 (Table 2). Reuterin was not detected in the postbiotics from any of these LAB strains (Table 1). The heatmap presents the correlation analysis between postbiotics and their bioactive profiling (Figure 2). Postbiotic S27 showed significantly higher concentrations of organic acids, particularly lactate and citrate, indicating a metabolically active profile. In contrast, postbiotic S42 exhibited an intermediate profile, combining high EPS production and protein content, suggesting interest for applications involving cell adhesion, mucosal protection, and some technological properties. However, the bioactive profile of postbiotic S62 suggests that it is particularly rich in proteins, polyphenols, and antimicrobial molecules, which is consistent with strong biotechnological potential.

### 2.2. Antimicrobial Activity of Postbiotic-CFS

**Figure 3:**
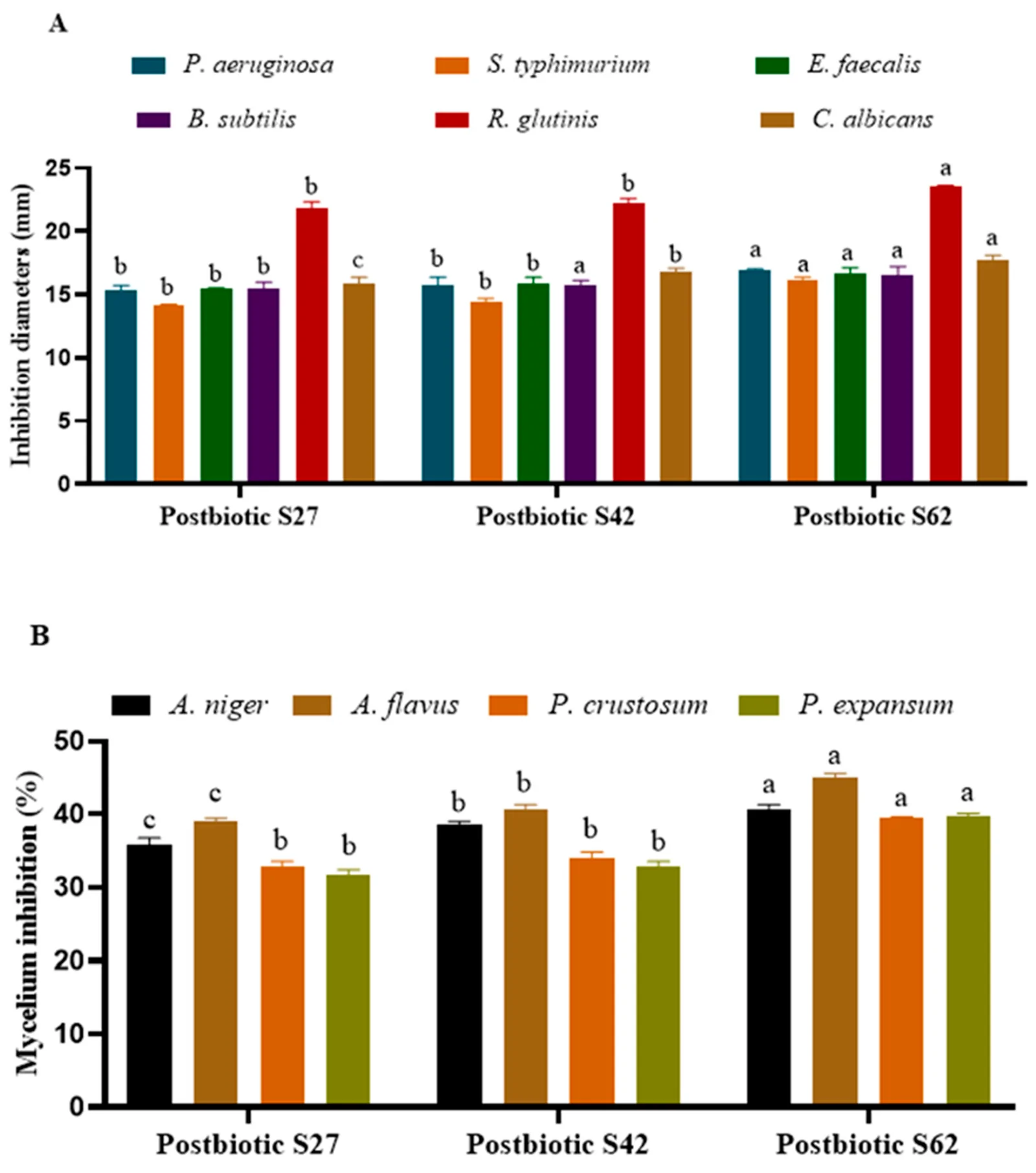
Antimicrobial activities of postbiotic-CFSs derived from *Levilactobacillus* and *Lactiplantibacillus* strains; Inhibition diameter (mm) (**A**); Mycelium inhibition (%) (**B**). ^a,b,c^ Means in the same postbiotic-CFS at each parameter with different lowercase letters differed significantly (*p* < 0.05).

**Figure 4:**
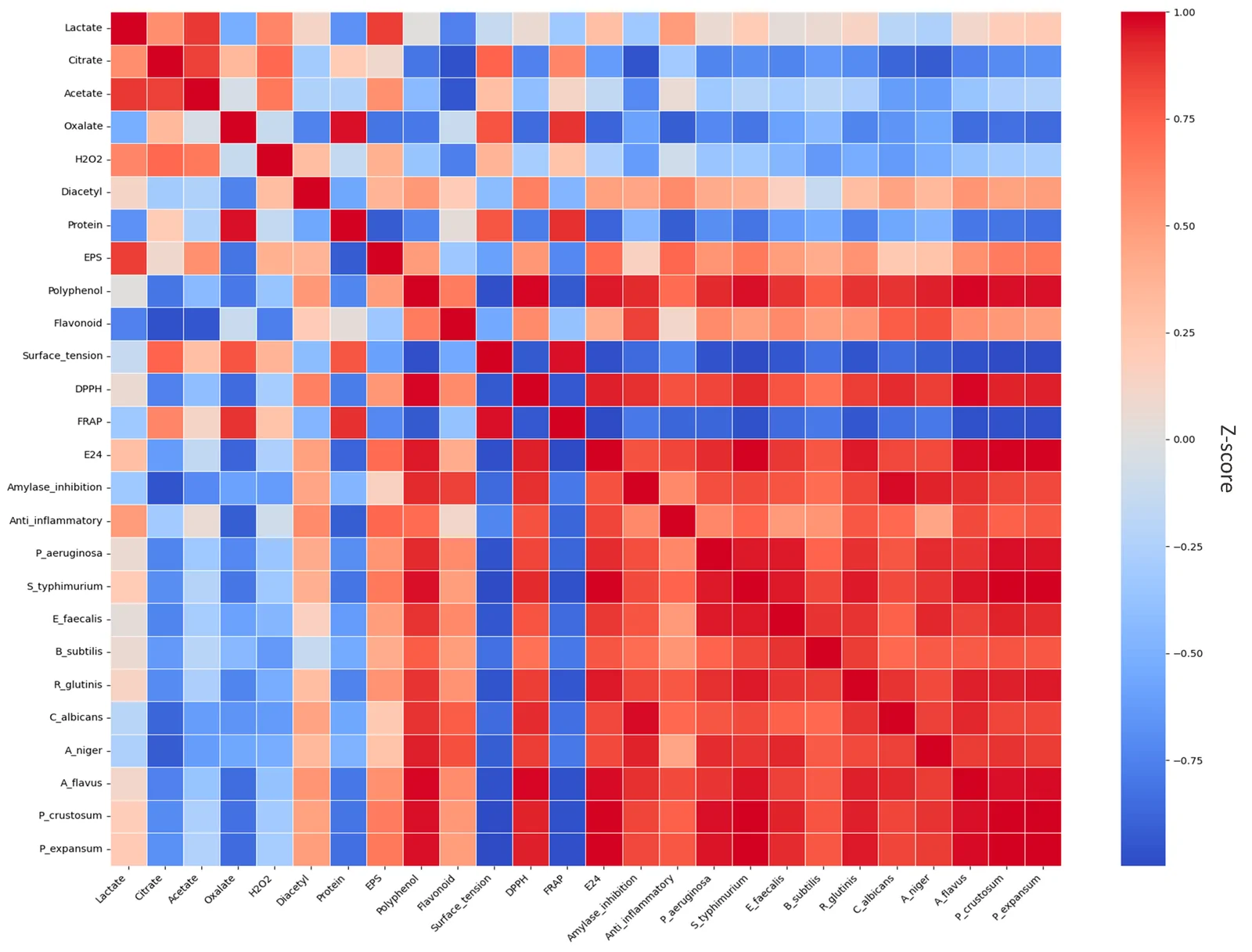
Pearson correlation matrix of bioactive profiling and functional properties of postbiotic-CFSs derived from three *Levilactobacillus* and *Lactiplantibacillus* strains.

The results of the antimicrobial activity of postbiotics against pathogenic and spoilage microorganisms are presented in Figure 3. All of the postbiotic-CFSs exhibited antibacterial activity against foodborne pathogenic bacteria, displaying inhibition zone diameters of 15.4–16.5 mm against *B. subtilis*, 15.4–16.7 mm against *E. faecalis*, 14.10–16.15 mm against *S. typhimurium*, and 15.30–16.9 mm against *P. aeruginosa* (Figure 3A). On the other hand, all postbiotics demonstrated high antifungal activity against *R. glutinis* and *C. albicans*, with the inhibition diameter ranging between 21.85 and 23.50 mm and 15.8 and 17.7 mm, respectively (Figure 3A). Moreover, the postbiotic-CFSs presented mycelium inhibition against *A. niger*, *A. flavus, P. crustosum*, and *P. expansum*, with the percentage ranging between 35.77 and 40.56%, 39.10 and 45.09%, 32.78 and 39.57%, and 31.67 and 39.70%, respectively (Figure 3B). Furthermore, the postbiotic-CFS S62 produced by *L. plantarum* S62 demonstrated the highest (*p* < 0.05) antimicrobial activity against pathogenic and spoilage microorganisms. In addition, postbiotic-CFS S62 exhibited significantly higher antimicrobial activity compared to S27 and S42 (Figure 2). These antimicrobial activities were positively associated with certain metabolites, highlighting their potential contribution to inhibitory effects against pathogenic and spoilage microorganisms (foodborne pathogenic bacteria, yeast, and molds) (Figure 4).

### 2.3. Antioxidant Activity of Postbiotic-CFS

**Figure 5:**
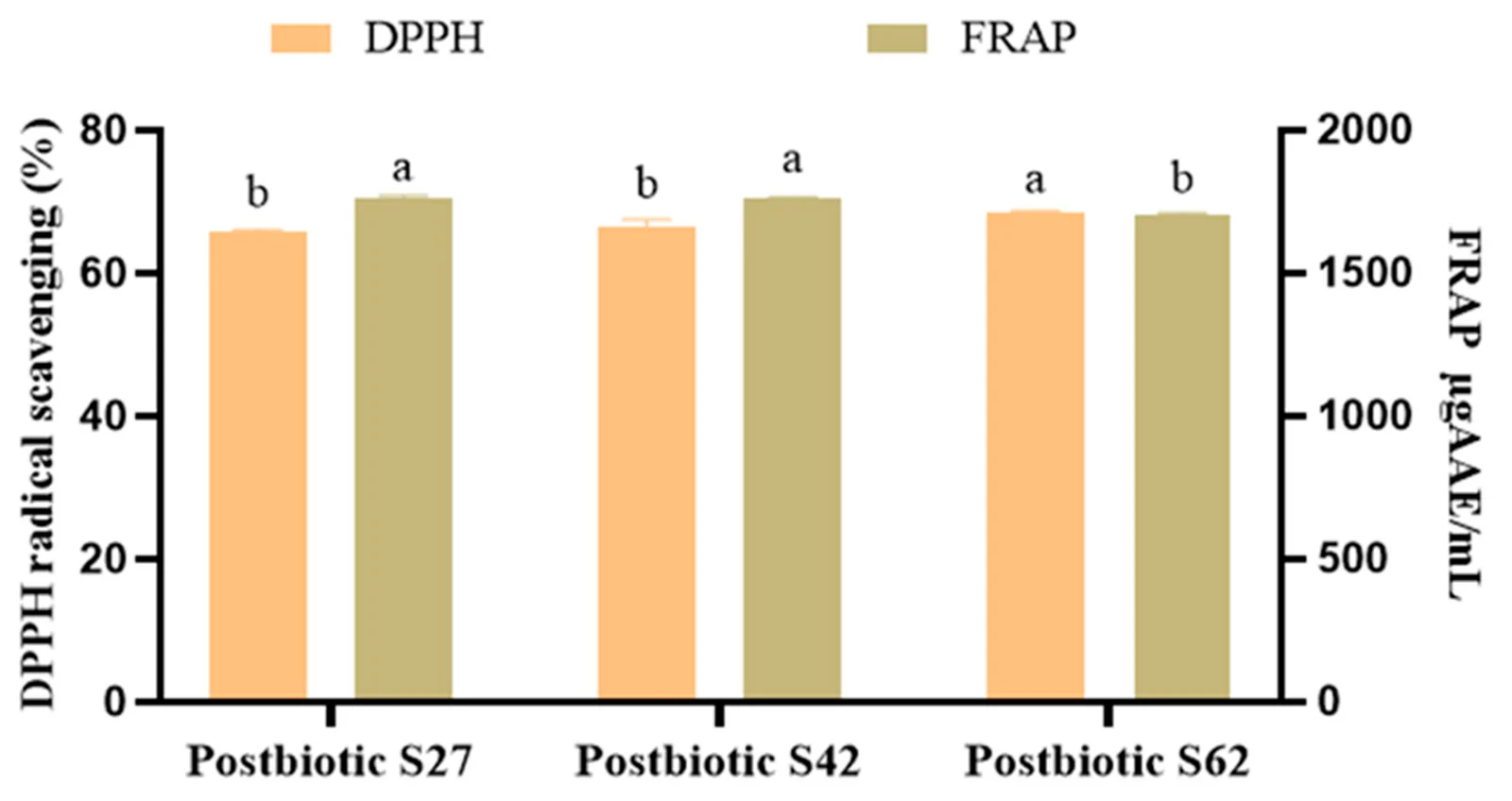
Antioxidant activities of postbiotic-CFSs derived from *Levilactobacillus* and *Lactiplantibacillus* strains. ^a,b^ Means in the same postbiotic-CFS at each parameter with different lowercase letters differed significantly (*p* < 0.05).

The results of DPPH and FRAP assays of the postbiotic-CFSs are presented in Figure 5. The DPPH radical scavenging activities were 65.91%, 66.63%, and 68.55% for postbiotic-CFSs S27, S42, and S62, respectively. The FRAP values of postbiotic-CFSs S27, S42, and S62 were 1765 µgAAE/mL, 1764.23 µgAAE/mL, and 1707.31 µgAAE/mL, respectively. Moreover, postbiotic-CFS S62 exhibited strong DPPH radical scavenging activity (68.55%) and was significantly higher (*p* < 0.05) compared to S27 and S42. However, postbiotic-CFSs S27 and S42 were significantly higher (*p* < 0.05) compared to S62 in terms of the FRAP assay. Furthermore, postbiotic-CFSs S27 and S62 exhibited strong antioxidant activities (DPPH and FRAP) (Figure 2) and positively correlated with polyphenol and flavonoid contents (Figure 4), suggesting a potential contribution of these metabolites to the antioxidant properties.

### 2.4. Anti-Inflammatory Activity of Postbiotic-CFSs

**Figure 6:**
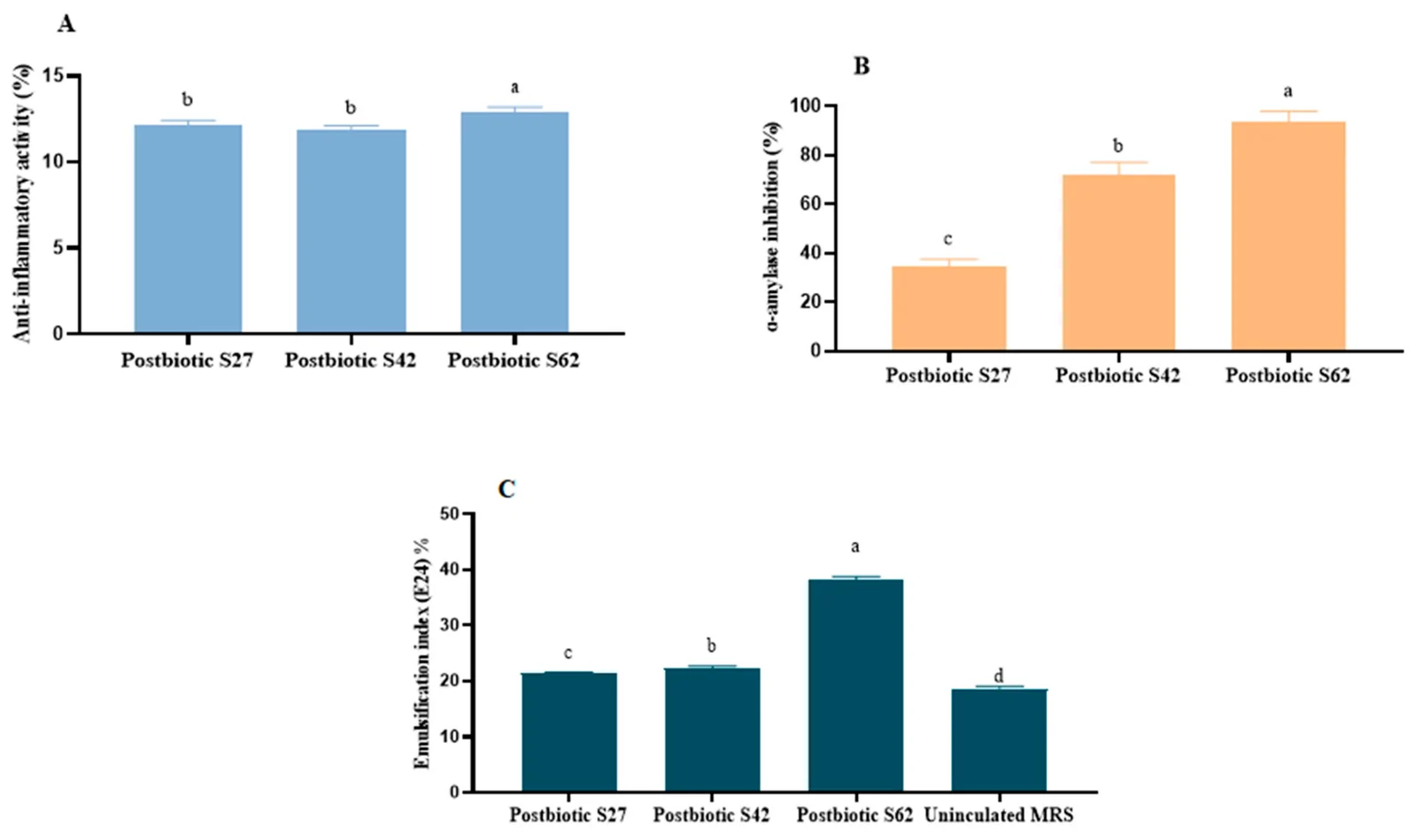
Functional properties of postbiotic-CFSs derived from *Levilactobacillus* and *Lactiplantibacillus* strains. Anti-inflammatory inhibition (**A**); α amylase inhibition (**B**); and emulsification index (E24%) (**C**). ^a–d^ Means in the same postbiotic-CFS at each parameter with different lowercase letters differed significantly (*p* < 0.05).

The anti-inflammatory activity of postbiotic-CFSs was evaluated by the inhibitory effect on protein (albumin) denaturation in vitro, and the results are summarized in Figure 6A. All of the postbiotic-CFSs from the LAB strains showed anti-inflammatory activity, with a percentage around 12%, and the anti-inflammatory activity of postbiotic-CFS S62 was significantly higher (*p* < 0.05) than that of postbiotics S27 and S42. Moreover, Figure 2 indicates that the molecules associated with the antioxidant effect could also contribute to the modulation of the inflammatory response.

### 2.5. Antidiabetic Activity of Postbiotic-CFSs

The in vitro antidiabetic potential of postbiotic-CFSs was evaluated using the α-amylase inhibition assay, and the results are presented in Figure 6B. All of the postbiotic-CFSs demonstrated significant α-amylase inhibition, with percentages of 34.38%, 71.88%, and 93.75% for postbiotic-CFSs S27, S42, and S62, respectively. Moreover, postbiotic-CFS S62 had significantly higher (*p* < 0.05) inhibition, with a high α-amylase inhibition of more than 93%.

### 2.6. Emulsification Index (E24%) Activity of Postbiotic-CFSs

The results of the emulsification index (E24%) activity of all of the postbiotic-CFSs are presented in Figure 6C. The E24% showed percentages of 21.5%, 22.3%, and 38.2% for postbiotic-CFSs S27, S42, and S62, respectively. Uninoculated MRS broth displayed an E24% of 18.75% attributable to Tween 80 content. Moreover, the emulsification index (E24%) of postbiotic-CFS S62 was significantly higher (*p* < 0.05) compared with S27 and S42 (Figure 6C). In addition, the emulsification index (E24%) showed a strong positive correlation with exopolysaccharide production, indicating its role in emulsification capacity. Conversely, surface tension showed a negative correlation with E24%, confirming biosurfactant activity (Figure 4).

### 2.7. Principal Component Analysis (PCA)

**Figure 7:**
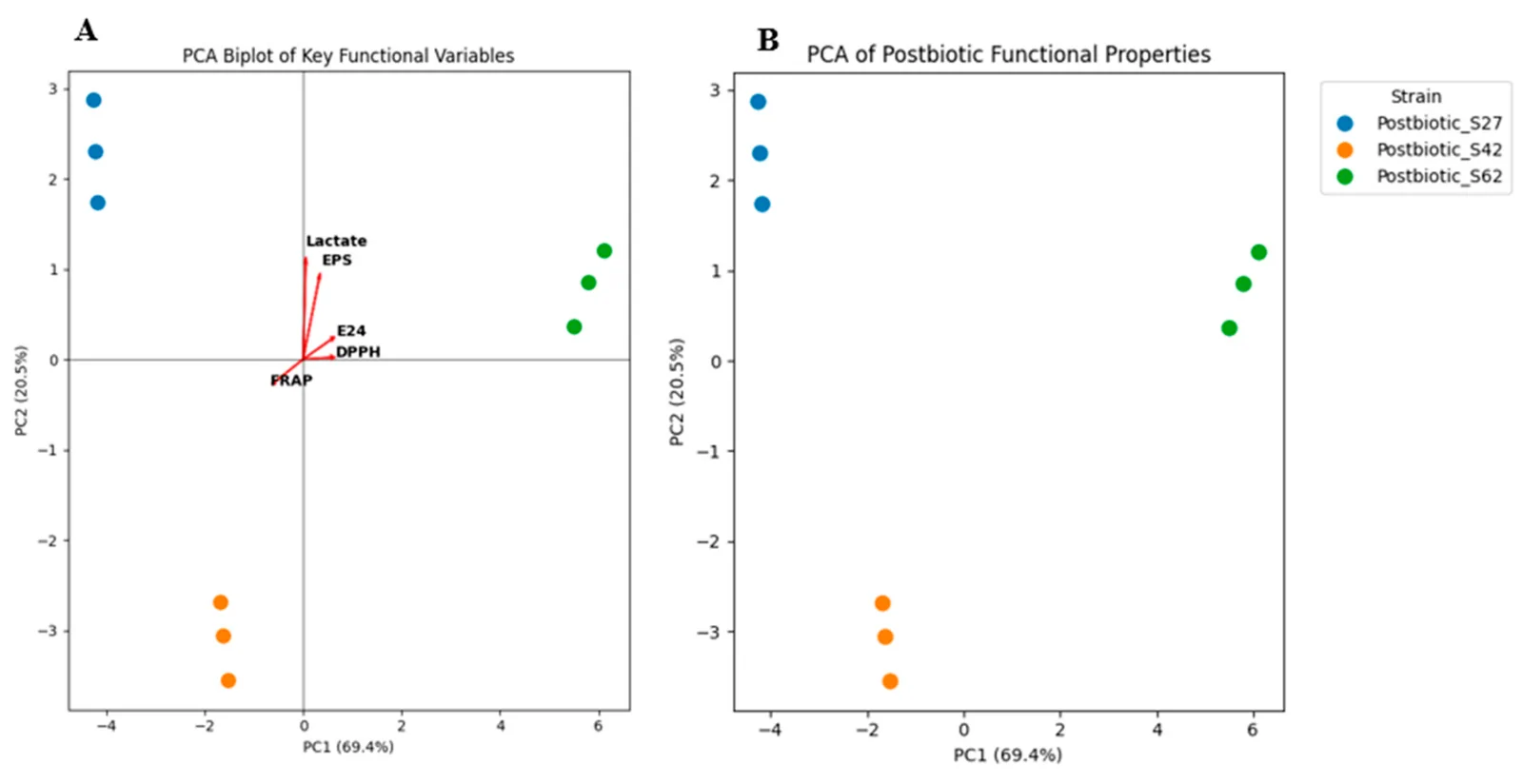
Principal component analysis (PCA) biplot illustrating the contribution of key functional variables (**A**) and the distribution of postbiotics based on their bioactive profiling and functional properties (**B**).

Principal component analysis (PCA) was performed to explore the overall variation and the relationships between the different functional properties of postbiotics and to highlight the similarities or differences between the three strains studied (postbiotic-CFS S27, postbiotic-CFS S42 and postbiotic-CFS S62) (Figure 7). While PCA explains most of the total variance, these multivariate statistical analyses were performed on a sample size of three strains. Consequently, these models serve primarily as exploratory comparative tools to highlight strain-specific profile clustering. PCA explains 89.9% of the total variance, with the first principal component (PC1) of 69.4% and the second component (PC2) accounting for 20.5%, indicating that these two components effectively summarize the variability of the data.

The biplot (Figure 7A) simultaneously represents the samples and the functional variables, thus providing a better understanding of the variables contributing to the observed separation among the postbiotic-CFSs. Postbiotic-CFS S62 was associated with lactate, DPPH, EPSs, and E24, which exhibited high values of these properties, while S42 showed higher FRAP activity. However, postbiotic-CFS S42 had lower overall organic acid levels, and showed a distinct profile enriched in proteinaceous components and total flavonoids, which are important in terms of overall functional properties. This finding highlights that postbiotic-CFS S27 is linked with fermentation metabolites and postbiotic-CFS S62 with EPSs, antioxidant and emulsifying properties, while S42 presents an intermediate functional profile.

The PCA score plot revealed a clear separation of the three postbiotic-CFSs along PC1 and PC2 (Figure 7B). Postbiotic-CFS S27 clustered in the positive PC2 region and was located on the opposite side of postbiotic-CFS S62, indicating that this postbiotic-CFS is primarily characterized by a relatively high amount of lactate and EPSs, with a lower contribution to DPPH and E24%, suggesting a focus on fermentative rather than antioxidant properties. Furthermore, postbiotic-CFS S62 clustered on the positive side for both PC1 and PC2 and was distinctly separated from S27 and S42, indicating substantial differences in its functional properties. This postbiotic is strongly associated with EPSs, lactate, antioxidant activities, and bioactive compounds. Thus, postbiotic-CFS S62 appears to have the most important functional profile. Conversely, postbiotic-CFS S42 occupied the negative region of both PC1 and PC2 and an intermediate position between the two strains, and this position reflects a less favorable functional profile, making it the least robust of the three studied. Consequently, these results indicate that the separation of strains in the PCA space is driven by differences in both bioactive profiling and functional properties.

## 3. Discussion

Postbiotic-CFSs derived from *Levilactobacillus* and *Lactiplantibacillus* strains showed the ability to produce organic acids and SCFAs, and this suggests that they are produced simultaneously during fermentative metabolism. Our findings were consistent with previous studies, which demonstrate that lactic acid was the predominant organic acid in postbiotic-CFSs, and the other organic acids and SCFAs were present in variable quantities [8,9,21]. In addition, SCFAs and organic acids exhibit various benefits in food products and have therapeutic potential, such as by contributing to the inhibition of pathogen proliferation, enhancing the aroma and flavor of fermented food products, and modulating systemic immune responses [7,21]. Moreover, our results show the presence of various bioactive metabolites with different concentrations. Previous studies demonstrate the presence of several bioactive compounds in postbiotics, including organic acids, fatty acids, protein compounds, vitamins, hydrogen peroxide alcohols, phenolic acids, and flavonoids [8,9,22], and the studies conducted by Ghasemi et al. [23] and Lara et al. [24] showed the ability of LAB to produce biosurfactants with a reduction of the surface tension mN/m. Overall, the presence of various metabolites in postbiotic-CFSs facilitates their application in several food products, enhancing their functional, sensorial, and bio-preservative properties, as well as in vitro therapeutic potential, while offering numerous health benefits for human consumers.

Postbiotic-CFSs exhibited high antimicrobial activities against foodborne pathogenic bacteria, yeast, and molds. These results agree with those of previous studies, which present the ability of postbiotic-CFSs to inhibit the growth of Gram-negative and Gram-positive pathogenic bacteria and biofilm formation [25,26,27]. Also, the postbiotic-CFS from *L. plantarum* exhibited antibacterial activity in vitro and in vivo against foodborne pathogens [28]. These findings demonstrate the possibility of applying postbiotic-CFSs as a therapeutic approach against infections and as an alternative to antibiotics, reducing the transmission of resistance genes to humans. Postbiotics were used to control opportunistic fungal spoilage and infections, promote anti-aflatoxigenic effects [17,29,30], and modulate the gut microbiota [31]. In our previous study focused on the antimicrobial activity of *Levilactobacillus* and *Lactiplantibacillus* strains, we demonstrate that the neutralization of CFS showed a reduction in antimicrobial activity, while the activity completely disappeared after treatment with proteolytic enzymes, which confirms the contribution of bioactive proteins to this antimicrobial activity [32]. Moreover, the antimicrobial activities of all of the postbiotic-CFSs were positively associated with several metabolites. Additionally, the antimicrobial activity of postbiotics (CFSs) is due to proteinaceous compounds acting in synergy with organic acids and other compounds [33]. Based on our previous findings regarding using CFSs against *R. glutinis* and *P. aeruginosa* [32], the inhibition diameters were aligned with the values reported in our previous study, and the antimicrobial spectrum was expanded in this study to include foodborne pathogens and spoilage microorganisms. In fact, the synergistic potential of all bioactive molecules present in postbiotic-CFSs contributes to inhibitory activity against various microorganisms, leading to the development of natural bio-preservatives and active food packaging to extend food shelf life.

All of the postbiotic-CFSs showed good antioxidant activities. The postbiotics demonstrated varied and strong antioxidant activities, including DPPH, FRAP, ABTS, and others [9,25,34]. Moreover, the antioxidant properties are related to the various bioactive metabolites, including total phenolic acid and total soluble sugar [34], short-chain fatty acids [35], exopolysaccharides [36], and bioactive peptides [37]. In the present study, the antioxidant activities of postbiotic-CFS S27 and S62 were linked to polyphenol and flavonoid contents (electron-donating bioactive metabolites), suggesting their contribution in vitro to radical-scavenging and reducing activities.

The inhibition of protein denaturation represents a standard preliminary screening for anti-inflammatory potential in vitro. Postbiotic-CFSs showed inflammatory properties through the inhibition of protein denaturation. Postbiotics exhibit in vitro functional potential by reducing inflammation in tissues and organs [38], and are used in the management of chronic inflammation [39]. Moreover, previous studies demonstrate good anti-inflammatory activity of postbiotics, and the inhibition of albumin denaturation has been related to a dose-dependent anti-inflammatory response [9,34]. Consequently, our postbiotic-CFSs exhibited a modest protective effect and were able to influence the immune response by reducing inflammation.

Postbiotics demonstrate antidiabetic potential through the inhibition of α-amylase activity. The α-amylase enzyme is associated with carbohydrate hydrolysis, releasing glucose into the bloodstream, and inhibiting its function is necessary to regulate blood sugar levels. Postbiotics present the greatest inhibition of α-amylase activity, and this activity is due to bioactive peptides [40,41]. In the present study, the correlation between different bioactive molecules and α-amylase inhibition suggests an interaction between enzymatic activities and bioactive metabolite production. These findings demonstrate in vitro inhibition of α-amylase, suggesting potential for delaying carbohydrate digestion and supporting the use of postbiotic-CFSs as a natural alternative in vitro for controlling diabetes and as an antidiabetic agent in the formulation of pharmaceutical products.

The results showed that all postbiotic-CFSs presented emulsification activity, and the uninoculated MRS showed an E24% of 18.75% and a surface tension of 40.65 mN/m. Various studies demonstrate that emulsification activity, measured by the emulsification index (E24%), is linked to biosurfactant and exopolysaccharide production [24,42]. These findings exhibited the potential uses of these postbiotic-CFSs rich in EPSs and biosurfactants as emulsifying agents in the formulation of food products.

## 4. Materials and Methods

### 4.1. Probiotic Lactic Acid Bacteria Strains

Three lactic acid bacteria *(Levilactobacillus brevis* S27, *Lactiplantibacillus pentosus* S42, and *Lactiplantibacillus plantarum* S62) were used to produce postbiotic-CFSs. These strains were previously isolated from fermented green olives and characterized for their probiotic properties [43] and maintained in De Man, Rogosa, and Sharpe (MRS) medium (Biokar, France) at −20 °C, supplemented with 20% (*v*/*v*) glycerol. They were cultured in MRS medium at 37 °C for 48 h before use.

### 4.2. Production and Preparation of Postbiotic Cell-Free Supernatants (Postbiotic-CFSs)

Postbiotic cell-free supernatants (postbiotic-CFSs) were obtained from LAB fermentations in MRS broth after 48 h at 37 °C. Following the incubation period, postbiotics were prepared and obtained by centrifugation of the culture at 10,000× *g* for 10 min at 4 °C, and then filter sterilization was performed using 0.22 μm pore size membrane filters (MF-Millipore, Merck, Darmstadt, Germany). The final pH values recorded were 3.95 for postbiotic-CFS S27, 3.9 for S42, and 3.8 for S62.

### 4.3. Profiling of Bioactive Metabolites from Postbiotic-CFSs

#### 4.3.1. Organic Acids and Short-Chain Fatty Acid (SCFA) Quantification by HPLC

The short-chain fatty acids were determined in the sterilized postbiotics from LAB strains using an HPLC system (Waters, Milford, MA, USA) equipped with a 1525 binary pump, 2489 refractometer, and Rezex-ROA-Organic acid H+ column (7.8 mm × 30 cm, Phenomenex, Torrance, CA, USA). Twenty microliters of the CFS was injected into the HPLC, and the elution was carried out at 0.6 mL/min using 0.005 N sulfuric acid at 40 °C. The calibration curves for lactate, acetate, citrate, butyrate, malate, and oxalate were used as standards, and the analysis was conducted in triplicate.

#### 4.3.2. Hydrogen Peroxide Quantification

Analysis of hydrogen peroxide in postbiotics was determined using the method of the AOAC [44]. The mixture of postbiotics with diluted sulphuric acid in equal volumes of 25 mL was titrated with potassium permanganate (0.1 M) until discoloration. Each milliliter of potassium permanganate was equivalent to 1.070 mg of hydrogen peroxide production, and uninoculated MRS broth was used as a control. The test was carried out in triplicate.

#### 4.3.3. Diacetyl Quantification

The production of diacetyl in postbiotics was determined according to the method of the AOAC [44]. First, 25 mL of postbiotics was mixed with 7.5 mL of hydroxylamine solution (1 M). The mixture was titrated with 0.1 M HCl, using bromophenol blue as an indicator, to a greenish-yellow endpoint. The equivalence factor of HCl to diacetyl is 21.52 mg, and uninoculated MRS broth was used as a control. The experiment was conducted in triplicate.

#### 4.3.4. Protein Contents

The protein content was estimated using the Bradford method [45]. A mixture of 200 µL of Bradford reagent (kit of Bio-Rad, Hercules, CA, USA) and 800 µL of each postbiotic sample was used to determine the protein concentrations at 595 nm, and uninoculated MRS broth was used as a control. Bovine serum albumin (Sigma-Aldrich, St. Louis, MO, USA) was used as a standard to determine the protein concentration in the samples. The experiment was conducted in triplicate.

#### 4.3.5. Reuterin Quantification

Reuterin production in postbiotics was determined using the colorimetric method of Circle, et al. [46]. The mixture of 1 mL of each sample of postbiotics and 0.75 mL of a 10 mM tryptophan solution (dissolved in 0.05 M HCl) was added to 3 mL of 37% HCl and incubated at 37 °C for 20 min. Acrolein (Sigma-Aldrich) was used as a standard solution and prepared in MRS broth medium with concentrations ranging from 0.05 to 6 mM. The absorbance of all samples was recorded at 560 nm, and the measurement was evaluated in triplicate.

#### 4.3.6. Exopolysaccharide (EPS) Quantification

Quantification of exopolysaccharide production in postbiotics was conducted using the protocol of Wu, et al. [47], with some modifications. The postbiotics were treated with a 4% (*v*/*v*) solution of 85% trichloroacetic acid (TCA) to remove the proteins. After centrifugation at 4 °C at 10,000 rpm for 30 min at 4 °C, EPSs were precipitated by adding twice the amount of absolute cold ethanol and maintaining it at 4 °C for 24 h, followed by 30 min of centrifugation at 4 °C, 10,000 rpm. The pellet was then redissolved in distilled water. EPS production was quantified by measuring the mass of lyophilized powder obtained per liter of culture medium (mg/L). Uninoculated MRS broth was used as a control, and all tests were conducted in triplicate.

#### 4.3.7. Total Phenolic and Flavonoid Contents

The total phenolic content of postbiotics was determined in samples using the Folin-Ciocalteu method [48] and was expressed in Gallic acid equivalents, using a Gallic acid (20–500 µg/mL) standard calibration curve. The total flavonoid content was determined using the aluminum chloride colorimetric method using Quercetin (5–250 µg/mL) as a standard, and the results were expressed as µg QuE/mL [49]; uninoculated MRS broth was used as a control. All tests were performed in triplicate.

#### 4.3.8. Biosurfactants Analysis

The biosurfactant production of postbiotics was evaluated using different qualitative tests. The tests included drop collapse, oil dispersion, Parafilm M, and microliter plates [23,50]. Uninoculated MRS broth without Tween 80 was used as a control, and all tests were performed in triplicate.

### 4.4. Antimicrobial Activity of Postbiotic-CFSs

#### 4.4.1. Antimicrobial Activity Using the Well Diffusion Assay

The antimicrobial activity of postbiotics derived from *Levilactobacillus* and *Lactiplantibacillus* strains was determined using a well diffusion assay. For this assay, 10^5^ CFU/mL of pathogenic bacteria (*Salmonella typhimurium*, *P. aeruginosa*, *Enterococcus faecalis*, or *Bacillus subtilis*) or yeast (*Rhodotorula glutinis* UPM22 or *Candida albicans*) was evenly spread on Mueller–Hinton (MH) broth (Biokar, France) or YEGP broth. The wells, cut in the agar using a sterile filter pipette tip, were charged with 100 μL of the postbiotic CFS, and the 1 mg/mL each of gentamicin and cycloheximide were used as controls against bacteria and yeast respectively. After incubation for 24 h at 37 °C/25 °C for bacteria and yeast, respectively; the inhibition zones were determined in mm. All tests were conducted in triplicate.

#### 4.4.2. Antifungal Activity

The antifungal activity of postbiotics was evaluated by the mycelium inhibition of fungi (*Aspergillus niger*, *Aspergillus flavus*, *Penicillium crustosum*, and *Penicillium expansum*). For this purpose, 10% (*v*/*v*) of each postbiotic-CFS derived from the LAB strains was added to the PDA medium and inoculated with 10^5^ spores/mL of fungi, and the medium with 10% (*v*/*v*) of uninoculated MRS broth was used as a control. All tests were incubated at 25 °C for 5 days, and the mycelium inhibition was calculated in triplicate using the following formula:

Mycelium inhibition (%) = (1 − (Fungal colony diameter (mm) in assay)/(Fungal colony diameter (mm) in control)) × 100

### 4.5. Antioxidant Activity of Postbiotic-CFSs

#### 4.5.1. DPPH Radical Scavenging Assay

The free radical-scavenging capacity of the postbiotic was evaluated using the 2,2-diphenyl-1-picrylhydrazyl (Sigma-Aldrich, USA) assay with gallic acid as the standard. Briefly, 100 µL of postbiotic was mixed with 1.0 mL of a 0.2 mM DPPH solution prepared in methanol. The mixtures were incubated at room temperature in the dark for 30 min, and the absorbance was measured at 517 nm. Uninoculated MRS broth was used as a control, using ascorbic acid (25–1000 µg/mL) as a standard. DPPH radical scavenging activity was performed in triplicate and calculated as the percentage inhibition.

DPPH radical scavenging (%) = (1 − (Absorbance of sample − Absorbance of blank)/(Absorbance of control)) × 100

#### 4.5.2. Ferric Reducing Antioxidant Power (FRAP) Assay

The ferric reducing antioxidant power (FRAP) assay of all postbiotics was performed following the method described by Ghabbour et al. [51], with some modifications. First, 1 mL of each postbiotic was mixed with 3 mL FRAP reagent, incubated in the dark for 4 min, and the absorbance was measured at 700 nm. The results of the FRAP assay were expressed as µg of ascorbic acid equivalents per milliliter of solution (µgAAE/mL) using ascorbic acid (25–1000 µg/mL) as a standard, and uninoculated MRS broth was used as a control. All experiments were performed in triplicate.

### 4.6. Anti-Inflammatory Activity of Postbiotic-CFSs

The anti-inflammatory activity of the postbiotics was performed in vitro using the protein (albumin) denaturation inhibitory activity described by Tarannum et al. [52], with some modifications. First, 1 mL of each postbiotic was mixed with 1 mL of BSA (1% in Tris-HCl 0.05 M, pH 6.6) and incubated in a 37 °C water bath for 15 min, followed by an additional incubation at 70 °C for 5 min. After cooling on ice for 5 min, the absorbance of the mixture was recorded at 660 nm. The calibration curve of diclofenac sodium (25–400 µg/mL) was used as a standard, and uninoculated MRS broth was used as a control. The percentage of protein denaturation inhibition was determined in triplicate and calculated using the following formula:

Anti-inflammatory activity (%) = (1 − (Absorbance of sample − Absorbance of blank)/(Absorbance of control)) × 100

### 4.7. Antidiabetic Activity of Postbiotic-CFSs

The antidiabetic activity of the postbiotics was evaluated using α amylase inhibitory activity, with minor modifications [40]. Briefly, 250 μL of postbiotics was added to 250 μL of 0.1 M PBS (containing 0.5 mg/mL of α-amylase solution, pH 6.4) and incubated at 25 °C for 10 min. Then, 250 μL of starch solution (1% *w*/*v* in 0.02 M sodium phosphate buffer) was added to the reaction mixture and incubated at 25 °C for 10 min. The reaction mixture was stopped using 500 μL of the DNS reagent incubated in a boiling water bath for 5 min. The reaction mixture was then cooled, and 10 mL of distilled water was added. The calibration curve of Acarbose (25–500 µg/mL) was used as a standard and uninoculated MRS broth was used as a control. The absorbance was measured at 540 nm, and the percent inhibition was calculated as follows:

α-amylase Inhibitory Activity (%) = (1 − (Absorbance of sample − Absorbance of blank)/(Absorbance of control)) × 100

### 4.8. Emulsification Index (E24%) of Postbiotic-CFSs

The Emulsification activity of postbiotics was detected by the addition of 2 mL of motor oil to an equal volume of each postbiotic, mixed with a vortex for 2 min, and allowed to stand for 24 h. Distilled water was used as a negative control and the uninoculated MRS as a positive control. The E24% was calculated by measuring the height of the emulsified layer (mm) using the following formula:

Emulsification index (E24%) = ((Height of the emulsion layer)/(Total height)) × 100

### 4.9. Statistical Analysis

Statistical analyses were conducted via Python (v3.12) followed by one-way ANOVA, and the GraphPad Prism 8 software (San Diego, CA, USA) was used to generate the figures. The data were displayed as means ± standard deviation, and Tukey’s HSD post hoc test was applied, which compared the means with a significant difference (*p* < 0.05).

## 5. Conclusions

This study investigates the bioactive profiling and in vitro multifunctional properties of postbiotic cell-free supernatants produced by three LAB strains. The postbiotic-CFSs obtained from three different probiotics *Levilactobacillus brevis* S27, *Lactiplantibacillus pentosus* S42, and *Lactiplantibacillus plantarum* S62, demonstrate various bioactive molecules. The evidence of antimicrobial, antioxidant, anti-inflammatory, antidiabetic, and emulsifying activities of these postbiotics highlights their multipurpose applications across various domains. Therefore, these postbiotic-CFSs are suitable for use as a natural biopreservative agent in food products and could be used in functional foods to improve consumer health and as next-generation therapeutics. However, further studies (e.g., in vivo) are needed to confirm the beneficial effects and overall properties of postbiotic-CFSs for food and therapeutic applications.

## Figures and Tables

**Table 1:** Profiling of bioactive metabolites in postbiotic-CFSs derived from *Levilactobacillus* and *Lactiplantibacillus* strains.

Postbiotic-CFS	Hydrogen Peroxide (µg/mL)	Diacetyl (µg/mL)	Reuterin (mM)	Protein Content (µg/mL)	EPS (mg/L)	Polyphenols (µg GAE/mL)	Flavonoids (µg QuE/mL)
**Postbiotic S27**	0.0074 ^a^ ± 0.0004	0.43 ^a^ ± 0.19	nd	177.16 ^b^ ± 0.13	535.27 ^b^ ± 0.78	386.00 ^c^ ± 8.51	83.66 ^c^ ± 0.27
**Postbiotic S42**	0.0072 ^a^ ± 0.0002	0.44 ^a^ ± 0.21	nd	192.42 ^a^ ± 0.54	244.00 ^c^ ± 0.08	407.60 ^b^ ± 7.29	114.51 ^a^ ± 1.20
**Postbiotic S62**	0.0071 ^a^ ± 0.0001	0.59 ^a^ ± 0.28	nd	154.65 ^c^ ± 0.57	600.62 ^a^ ± 0.19	451.93 ^a^ ± 2.75	109.73 ^b^ ± 1.02

Values are the mean ± standard deviation (SD) of triplicates. ^a,b,c^ Means in the same column at each parameter with different lowercase letters differed significantly (*p* < 0.05). EPS: Exopolysaccharides. nd: not detected.

**Table 2:** Biosurfactant production in postbiotic-CFSs derived from *Levilactobacillus* and *Lactiplantibacillus* strains.

Postbiotic-CFS	Oil Displacement Test (mm)	Parafilm M Test	Drop-Collapse Test	Glass Slide Test	Surface Tension (mN/m)
**Postbiotic S27**	7.1 ^c^ ± 0.14	++	+	+	38.3 ^b^ ± 0.28
**Postbiotic S42**	8.2 ^b^ ± 0.28	++	+	+	37.45 ^c^ ± 0.49
**Postbiotic S62**	10.3 ^a^ ± 0.42	++	++	++	34.35 ^d^ ± 0.21
**Uninoculated MRS**	nd	-	-	-	40.65 ^a^ ± 0.15

(-): No production, (+): Low production, (++): Medium production. Values are the mean ± standard deviation (SD) of triplicates. ^a–d^ Means in the same column at each parameter with different lowercase letters differed significantly (*p* < 0.05). nd: not detected.

## Data Availability

Data will be made available upon request.

## Abbreviations

CFS	Cell-free supernatant
DPPH	2,2-diphényl-1-picrylhydrazyle
EPS	Exopolysaccharide
FRAP	Ferric reducing antioxidant power
GRAS	Generally recognized as safe
HPLC	High-performance liquid chromatography
ISAPP	International scientific association for probiotics and prebiotics
LAB	Lactic acid bacteria
SCFA	Short-chain fatty acids
